# NetworkCommons: bridging data, knowledge, and methods to build and evaluate context-specific biological networks

**DOI:** 10.1093/bioinformatics/btaf048

**Published:** 2025-02-05

**Authors:** Victor Paton, Denes Türei, Olga Ivanova, Sophia Müller-Dott, Pablo Rodriguez-Mier, Veronica Venafra, Livia Perfetto, Martin Garrido-Rodriguez, Julio Saez-Rodriguez

**Affiliations:** Institute for Computational Biomedicine, Heidelberg University Hospital and Heidelberg University, Heidelberg, 69120, Germany; Institute for Computational Biomedicine, Heidelberg University Hospital and Heidelberg University, Heidelberg, 69120, Germany; Institute for Computational Biomedicine, Heidelberg University Hospital and Heidelberg University, Heidelberg, 69120, Germany; Institute for Computational Biomedicine, Heidelberg University Hospital and Heidelberg University, Heidelberg, 69120, Germany; Institute for Computational Biomedicine, Heidelberg University Hospital and Heidelberg University, Heidelberg, 69120, Germany; Department of Biology and Biotechnologies “C. Darwin”, University of Rome La Sapienza, 00185 Rome, Italy; Ph.D. Program in Cellular and Molecular Biology, Department of Biology, University of Rome ‘Tor Vergata’, 00133 Rome, Italy; Department of Biology and Biotechnologies “C. Darwin”, University of Rome La Sapienza, 00185 Rome, Italy; Institute for Computational Biomedicine, Heidelberg University Hospital and Heidelberg University, Heidelberg, 69120, Germany; Molecular Systems Biology Unit, European Molecular Biology Laboratory (EMBL), Heidelberg, 69117, Germany; European Bioinformatics Institute (EMBL-EBI), Hinxton, Cambridgeshire, CB10 1SD, United Kingdom; Institute for Computational Biomedicine, Heidelberg University Hospital and Heidelberg University, Heidelberg, 69120, Germany; European Bioinformatics Institute (EMBL-EBI), Hinxton, Cambridgeshire, CB10 1SD, United Kingdom

## Abstract

**Summary:**

We present NetworkCommons, a platform for integrating prior knowledge, omics data, and network inference methods, facilitating their usage and evaluation. NetworkCommons aims to be an infrastructure for the network biology community that supports the development of better methods and benchmarks, by enhancing interoperability and integration.

**Availability and implementation:**

NetworkCommons is implemented in Python and offers programmatic access to multiple omics datasets, network inference methods, and benchmarking setups. It is a free software, available at https://github.com/saezlab/networkcommons, and deposited in Zenodo at https://doi.org/10.5281/zenodo.14719118.

## 1 Introduction

Network biology leverages computational methods to clarify complex interactions among molecules, including genes, proteins, and metabolites (Zitnik *et al.* 2023). In particular, context-specific network inference methods integrate context-agnostic prior knowledge with omics data to identify subnetworks associated with specific conditions, such as perturbations or diseases (Garrido-Rodriguez *et al.* 2022). These networks then enable various downstream applications, including omics data interpretation and identification of potential drug targets (Perrone *et al.* 2024).

A wide range of tools have been developed to infer networks from different combinations of knowledge and data. However, these tools do not share a common application programming interface (API). This lack of interoperability hinders the benchmarking of these methods as well as the reuse and combination of their modules.

Here, we present NetworkCommons, a Python package that unifies programmatic access to (i) prior knowledge, (ii) omics data, and (iii) network contextualization methods. With these components, we provide a platform for users to create, apply, and evaluate custom subnetwork identification problems. The value of this platform is illustrated by providing access to four preprocessed omics datasets, eight network inference methods, and four benchmarking setups, as a stepping stone toward a “Network commons”, an ecosystem of resources and methods for network biology. All code and extensive documentation are available at https://networkcommons.readthedocs.io/.

## 2 Results

**Figure 1.**
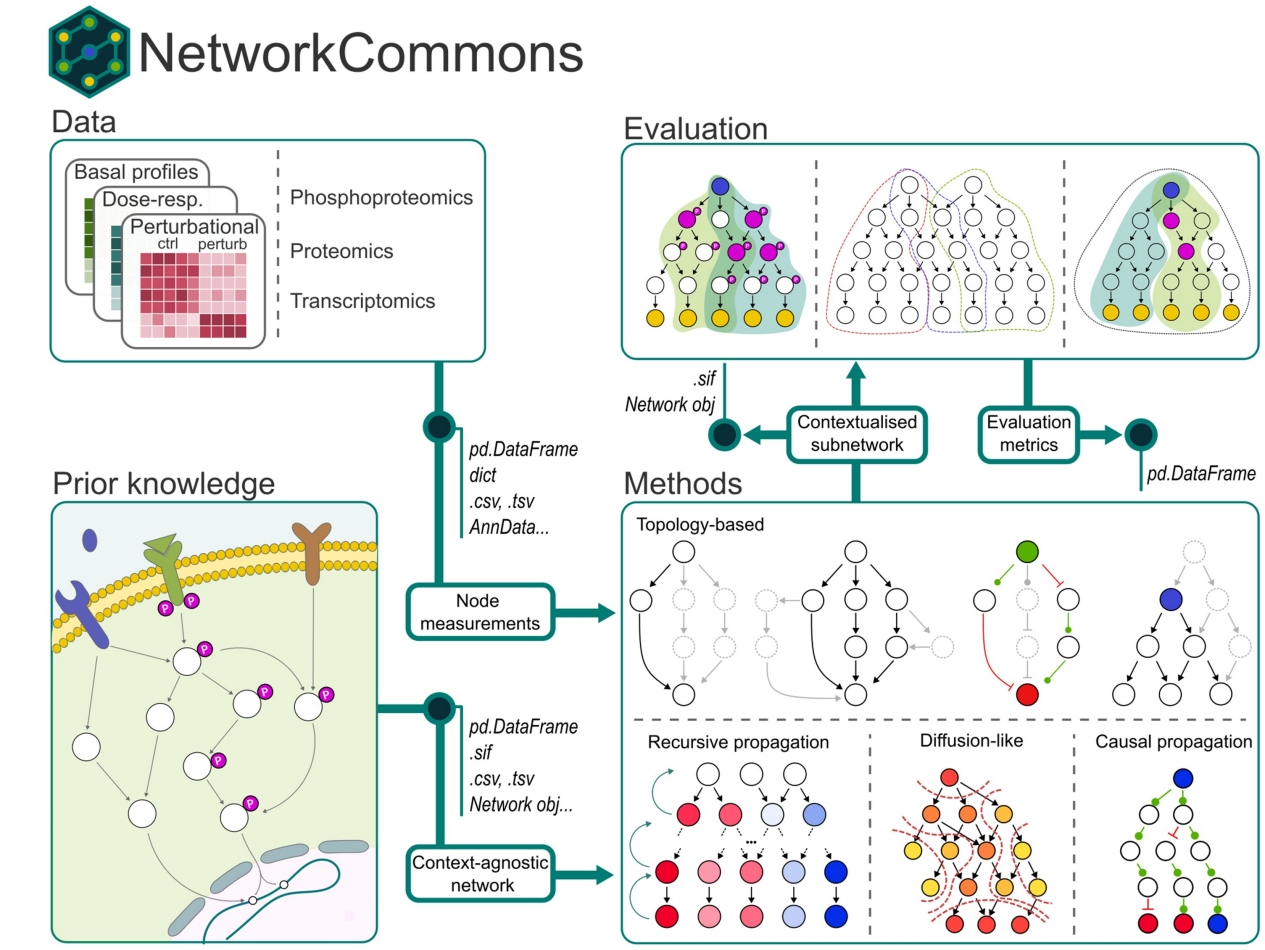
Overview of NetworkCommons. The package connects four different areas: data, prior knowledge, contextualization methods, and evaluation.

NetworkCommons offers a high-level API for accessing prior knowledge, omics data, and contextualization methods, allowing users to integrate, evaluate, and visualize generated subnetworks. The package is divided into four primary modules (Fig. 1).

The first module provides access to prior knowledge networks (PKNs), representing various types of context-independent, protein-level interactions. Users can either use one of the built-in PKNs or import their own by converting it into a NetworkCommons PKN object (Supplementary Data SIV.1). The currently built-in PKNs include only directed networks. Directed networks are of particular interest as they contain the minimal molecular information needed to generate causal hypotheses and statements (Touré *et al.* 2021). Of note, the API and some of the included methods are also fully compatible with undirected graphs. The networks may also include a weight interaction attribute to indicate up- or down-regulation. These weights can also convey other quantitative or qualitative information, although most of the network contextualization methods do not directly use this information. The built-in prior knowledge relies primarily on OmniPath (Türei *et al.* 2021), a database combining dozens of resources, covering protein–protein, kinase–substrate, ligand–receptor interactions, as well as gene regulatory networks. Other sources can be easily added (Supplementary Data SIV.1).

The omics data module provides built-in access to preprocessed omics datasets, encompassing diverse types of omics data across multiple biological contexts. We illustrate the access to four datasets, and also how users can connect their own datasets to the platform (Supplementary Data SIV.2). All datasets are managed through pandas data frames, with an additional interface available for AnnData objects from the scverse framework (Virshup *et al.* 2023, 2024), as illustrated in a dedicated vignette that uses perturbational data from pertpy (Heumos *et al.* 2024). The first among the datasets is dose-response phosphoproteomics data from DecryptM, as preprocessed by its authors (Zecha *et al.* 2023). Second, we curated and reprocessed data from the PANACEA DREAM challenge (Douglass *et al.* 2022), which profiles single-dose transcriptomic responses to 32 kinase inhibitors across 11 cancer cell lines. Third, we added basal metabolomics and transcriptomics data from the NCI-60 cancer cell line collection (Shoemaker 2006). Lastly, we provide access to harmonized multi-omics data from the Clinical Proteomic Tumor Analysis Consortium (Li *et al.* 2023), which includes genomics, transcriptomics, proteomics, and phosphoproteomics data for various tumor and adjacent normal tissue samples across a wide range of cancer types. This second module offers access to a variety of large datasets spanning basal, perturbed, and clinical contexts, and users are welcome to input their own datasets.

The methods module offers a consistent API for various network contextualization methods. Each method requires, at a minimum, a PKN object and a set of upstream and downstream measurements of molecular activities. The output of each method is a contextualized subnetwork. NetworkCommons already includes a range of contextualization methods, such as topology-based, recursive propagation, diffusion approaches, and causal propagation (described in detail in Supplementary Data SII). New methods can be included from external modules by wrappers, or implemented directly within NetworkCommons, using libraries already supported in the framework, such as NetworkX (Hagberg *et al.* 2008) or CORNETO (Rodriguez-Mier *et al.* 2024). For example, the causal reasoning method CARNIVAL (Liu *et al.* 2019), as implemented in CORNETO, is made available in NetworkCommons by a minimal wrapper (Supplementary Data SII.6). We also incorporate SignalingProfiler (Venafra *et al.* 2024), which integrates topology-based methods and CORNETO to generate hierarchical signaling models (Supplementary Data SII.7). NetworkCommons also provides network visualization and other plotting functions to support quality control, data exploration and result interpretation.

The fourth main component of NetworkCommons, the evaluation module, allows for the benchmarking of contextualization methods. We illustrate this with four strategies to evaluate context-specific subnetworks, using the datasets already available via the omics data module. Each evaluation strategy requires context specific networks as input, and outputs a set of evaluation metrics. The first evaluation strategy makes use of the perturbational profiles from compounds in the PANACEA project. Here, the networks are scored according to the number of recovered off-target proteins. The second strategy uses DecryptM data to recover phosphorylation changes based on sensitivity to drug perturbations. The third strategy scores subnetworks based on the enrichment of an a priori expected gene set. Lastly, we used harmonized data from the Clinical Proteomic Tumor Analysis Consortium to showcase our fourth evaluation strategy. Here, we used protein abundance from proteomics, and activity scores inferred from transcriptomics to contextualize networks. These are then evaluated based on the enrichment of kinase activities from phosphoproteomics. More details about the strategies are available in Supplementary Data SIII, about the implementation and creating new strategies, see Supplementary Data SIV.4.

## 3 Discussion

In this note, we present NetworkCommons, a platform for the integration of omics data and prior knowledge with methods to generate context-specific molecular interaction networks. With our design, we aim for interoperability, ease of access, and robustness of existing and future components. While the current version represents the beginning of a broader effort, we already illustrate its potential by providing integrated access to several data sources, methods, and different evaluation strategies for contextualization methods. We propose a programmatic environment where these evaluation strategies can be tested. We provide four of them to demonstrate this concept, and as a starting point for method developers to evaluate new approaches.

In the future, we aim to expand the ecosystem to include more datasets, methods, and prior knowledge, to provide support for a wider range of applications.

We plan to incorporate additional methods, such as phuEGO (Giudice *et al.* 2024), and to expand our networks to functional and disease annotations, leveraging integrated knowledge graphs (Lobentanzer *et al.* 2023). Additionally, we plan to extend the scope of available prior knowledge and methodologies to include undirected networks. This includes leveraging module discovery methods like DOMINO (Levi *et al.* 2021), which can provide valuable insights, such as enhancing our understanding of genetic variations (Barrio-Hernandez *et al.* 2023). We also aim to further develop the API by introducing higher level objects, expanding the use of AnnData for handling omics data (Virshup *et al.* 2023), open ways toward Boolean modeling (Ruscone *et al.* 2024), and improve the connection with other ecosystems such as scverse for single-cell data analysis and preprocessing, and in particular pertpy to leverage perturbational data (Heumos *et al.* 2024).

With NetworkCommons, we aim to create a meeting point for the network biology community. We envision it as a key infrastructure, modules, and utilities that everyone can reuse for their own developments and applications, and supports a broader collective effort to build robust and efficient context-specific network inference methods and advanced benchmarks. To facilitate this, we created contribution guidelines, inviting current and future developers within the network biology field to contribute with data, prior knowledge, methods and evaluation strategies. By providing a unified platform for data, prior knowledge, and methods, we aim not only to empower users to use mechanistic network approaches in their research, but also to encourage the community to join efforts and push the boundaries of network biology.

## Supplementary Material

btaf048_Supplementary_Data

## Data Availability

NetworkCommons is implemented in Python and offers programmatic access to multiple omics datasets, network inference methods, and benchmarking setups. It is a free software, available at https://github.com/saezlab/networkcommons, and deposited in Zenodo at https://doi.org/10.5281/zenodo.14719118. Additional information about omics data included and used in NetworkCommons can be found at https://networkcommons.readthedocs.io/en/latest/datasets.html
