# Supplementary figures and images for: NetworkCommons: bridging data, knowledge, and methods to build and evaluate context-specific biological networks
Source: Bioinformatics. 2025 Feb 5;41(2):btaf048. doi: 10.1093/bioinformatics/btaf048 (PMC11846666; doi:10.1093/bioinformatics/btaf048)

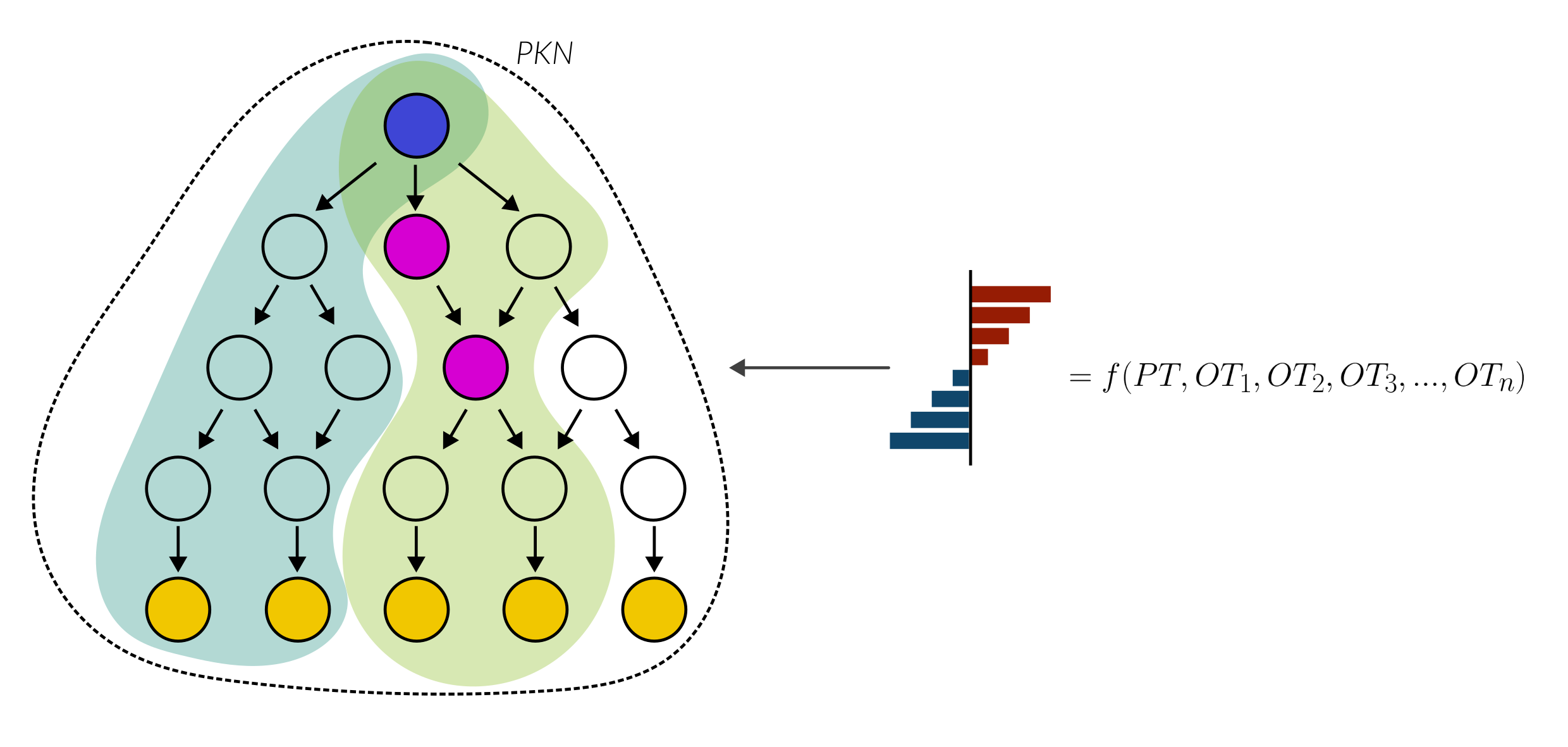

Supplement: btaf048_Supplementary_Data [file btaf048_supplementary_data.zip › supp6.png]

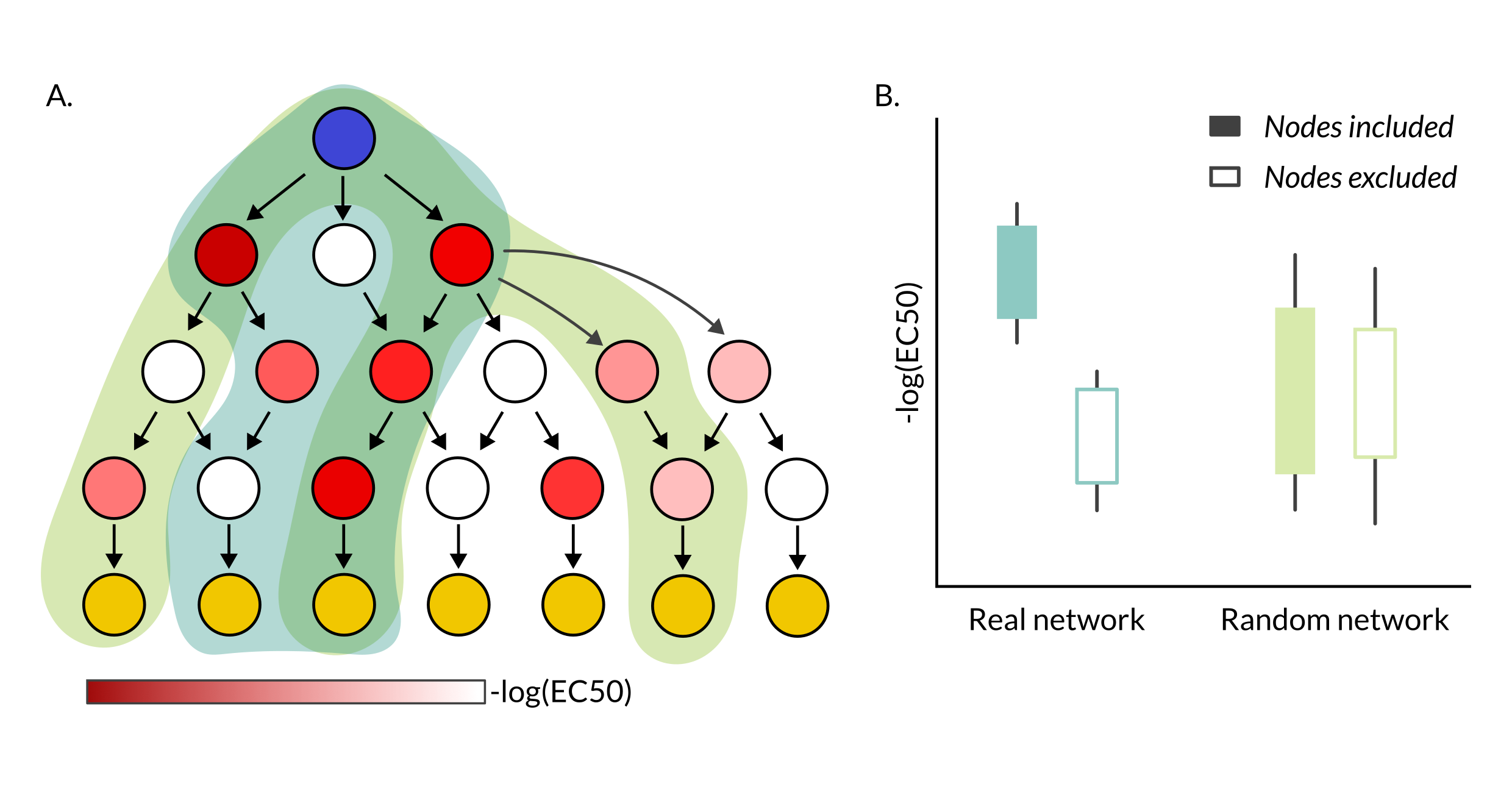

Supplement: btaf048_Supplementary_Data [file btaf048_supplementary_data.zip › supp7.png]

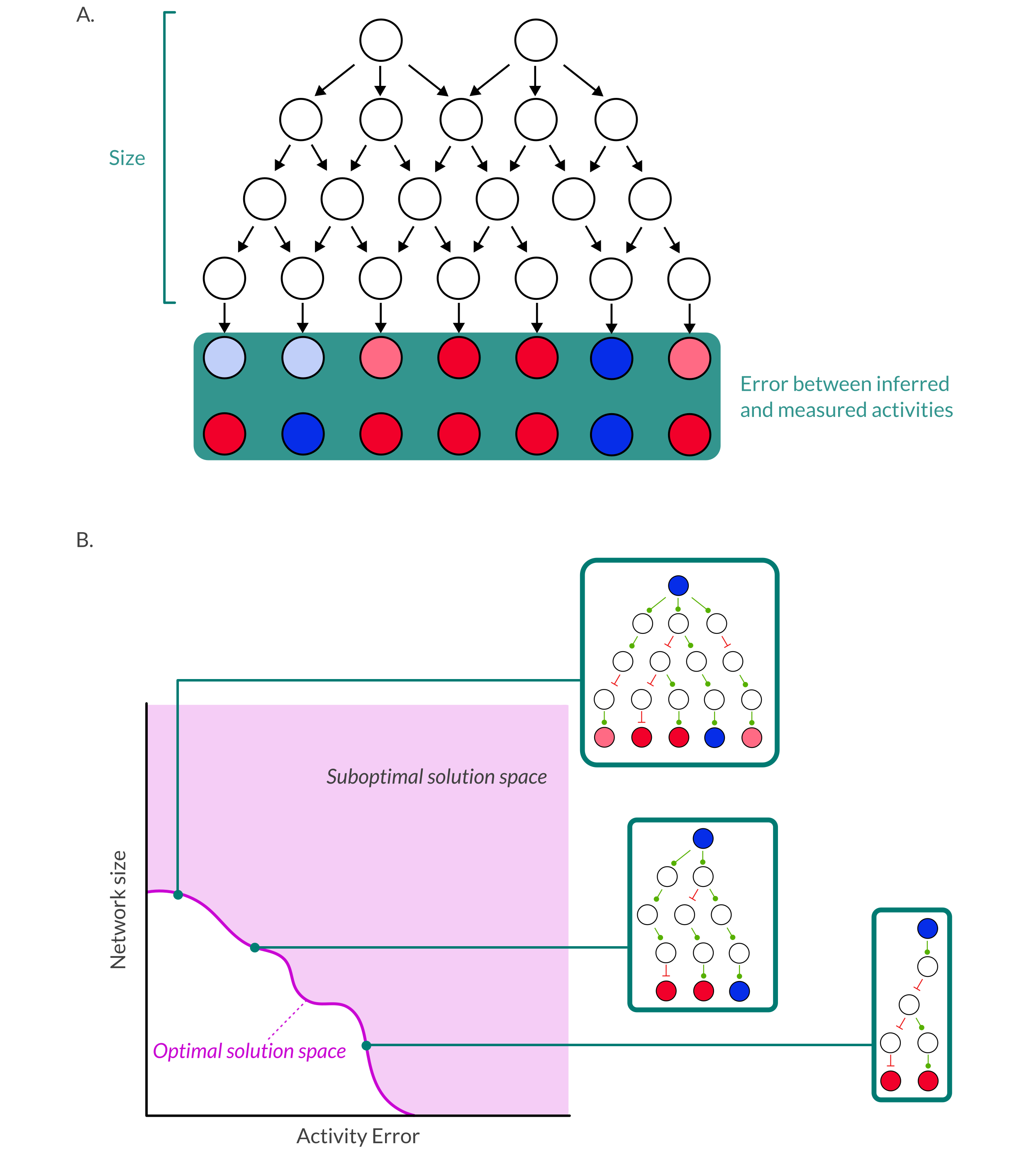

Supplement: btaf048_Supplementary_Data [file btaf048_supplementary_data.zip › supp2.png]

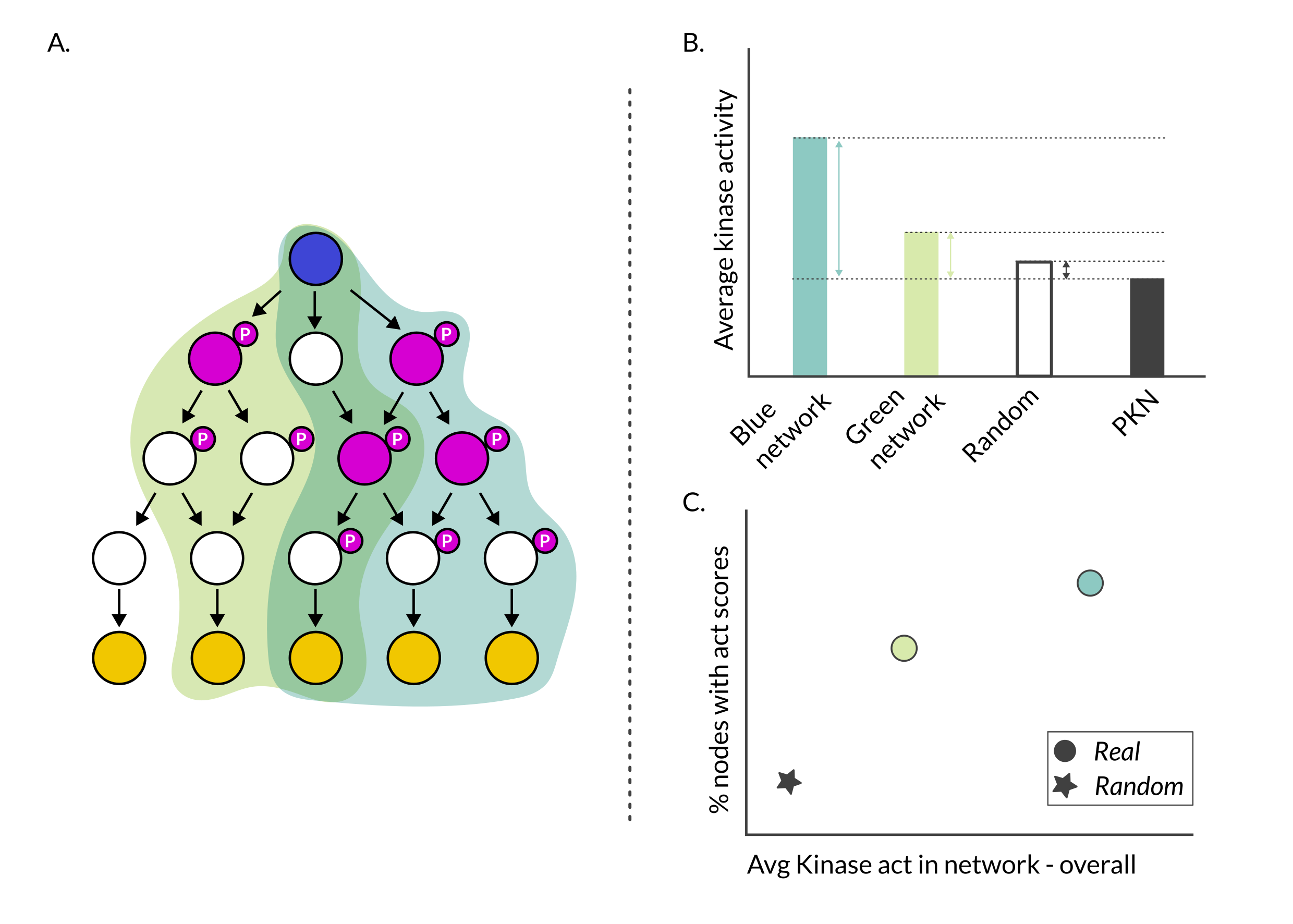

Supplement: btaf048_Supplementary_Data [file btaf048_supplementary_data.zip › supp8.png]

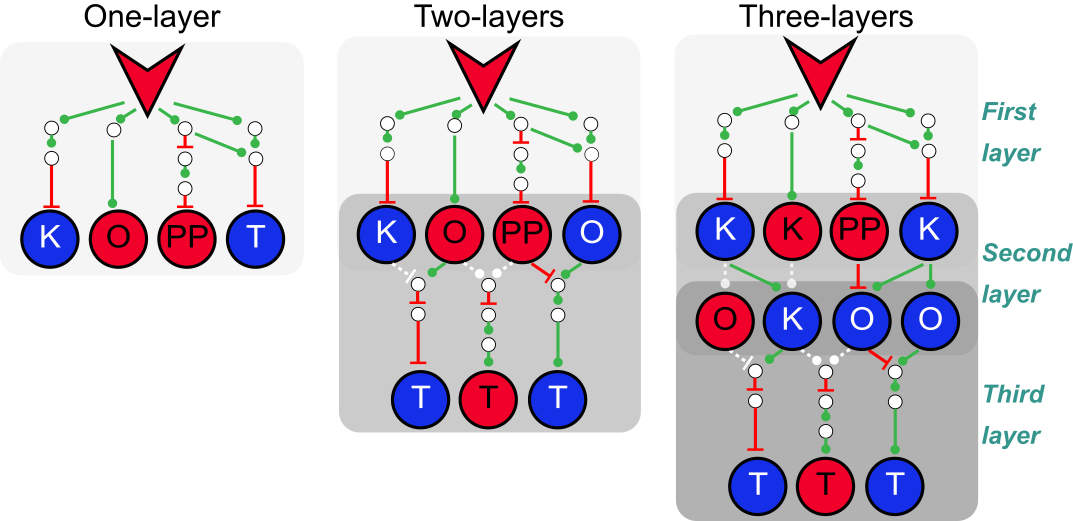

Supplement: btaf048_Supplementary_Data [file btaf048_supplementary_data.zip › supp4.png]

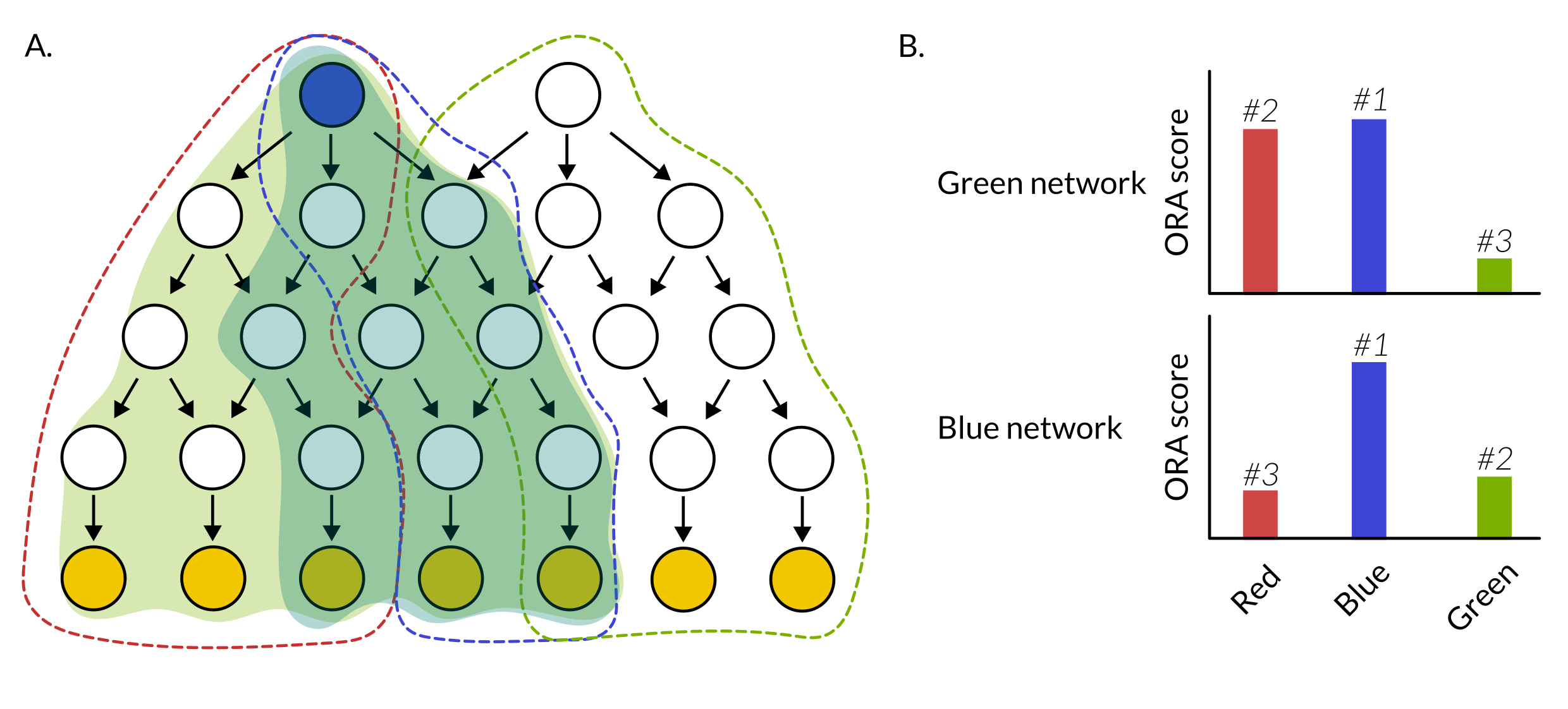

Supplement: btaf048_Supplementary_Data [file btaf048_supplementary_data.zip › supp5.png]

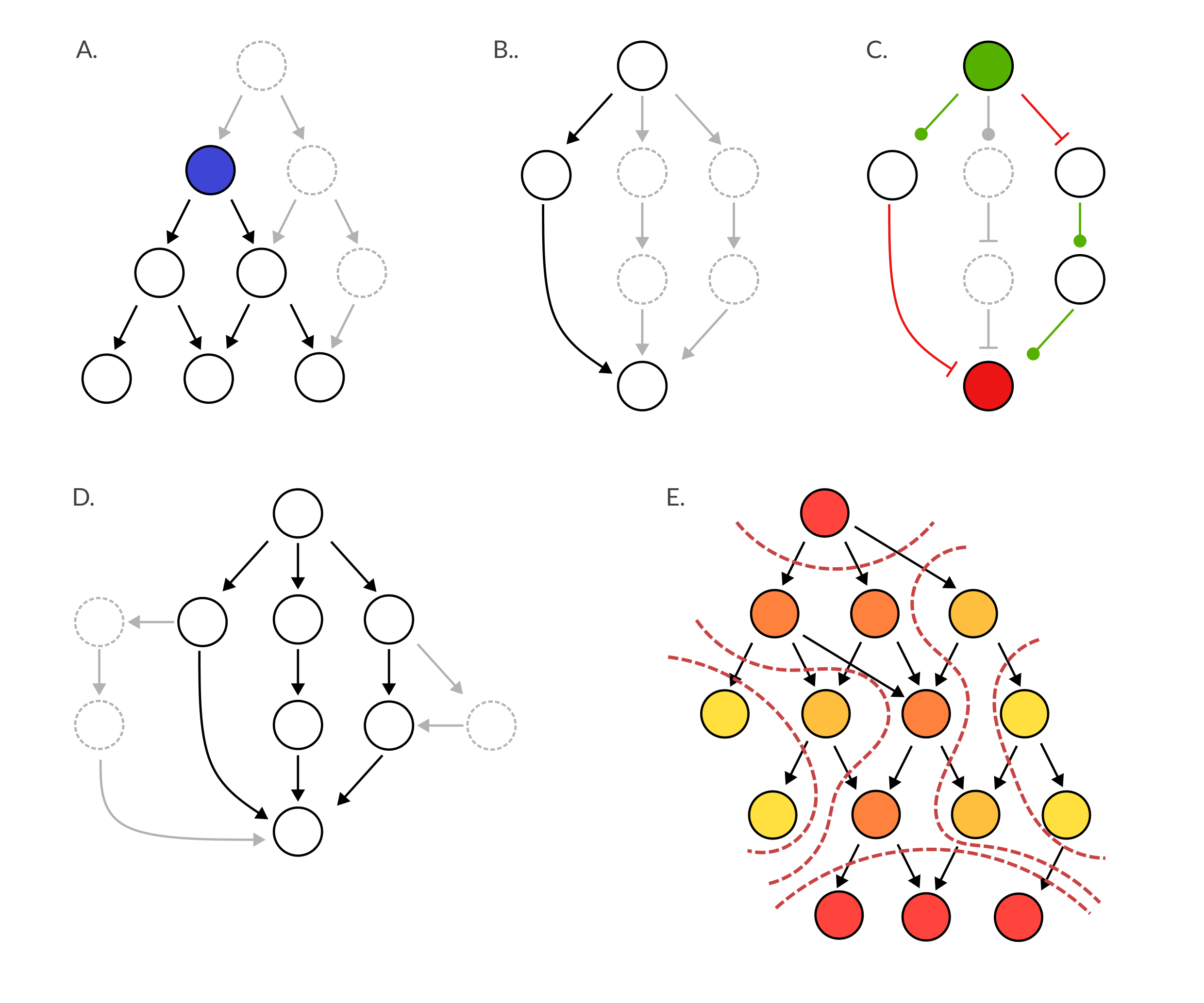

Supplement: btaf048_Supplementary_Data [file btaf048_supplementary_data.zip › supp1.png]

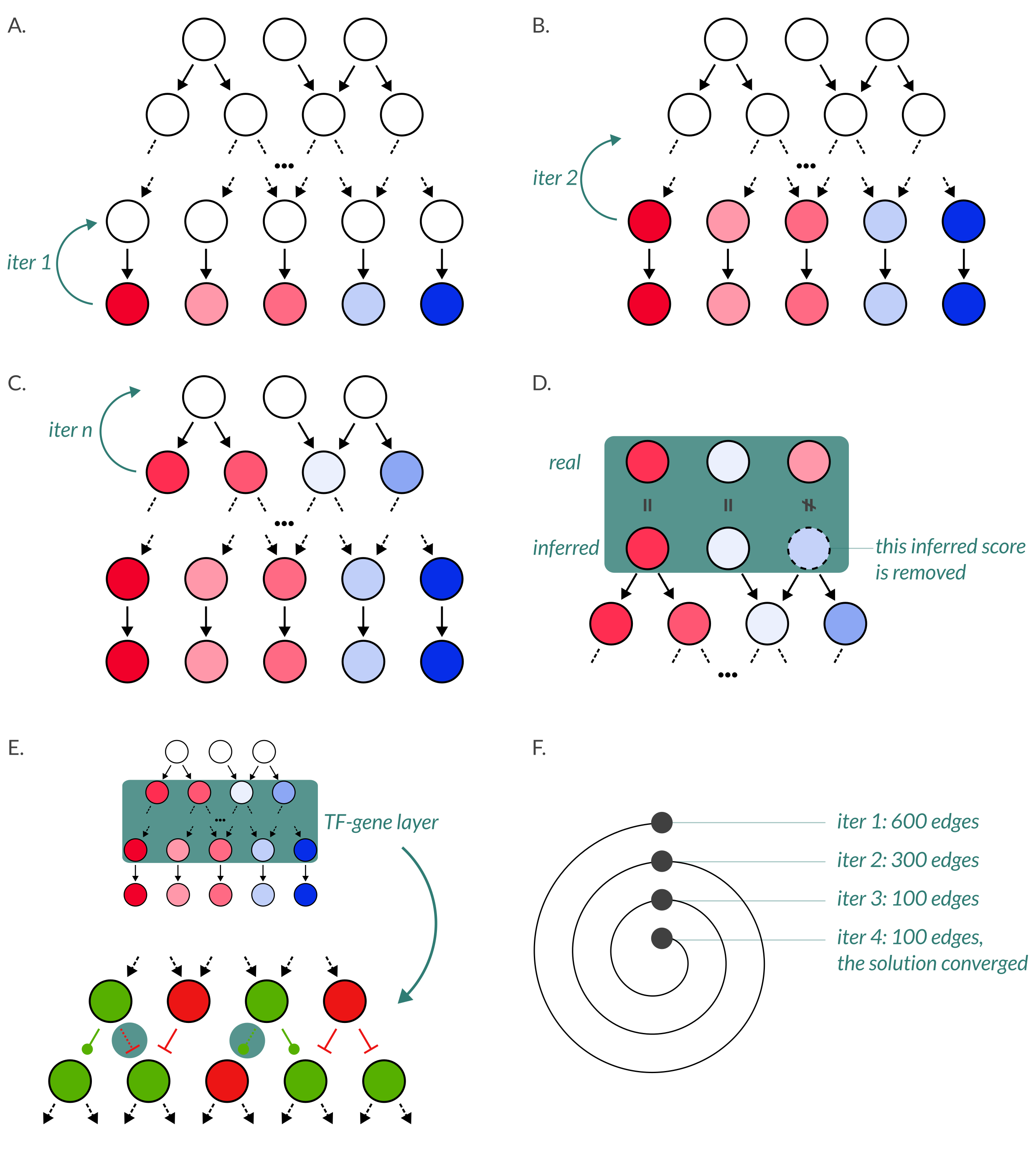

Supplement: btaf048_Supplementary_Data [file btaf048_supplementary_data.zip › supp3.png]
